# Supplementary material for: High-throughput screens using photo-highlighting discover BMP signaling in mitochondrial lipid oxidation
Source: Nat Commun. 2017 Oct 11;8:865. doi: 10.1038/s41467-017-00944-3 (PMC5636786; doi:10.1038/s41467-017-00944-3)
Supplement: Supplementary file 1 — Supplementary Information [file 41467_2017_944_MOESM1_ESM.pdf]

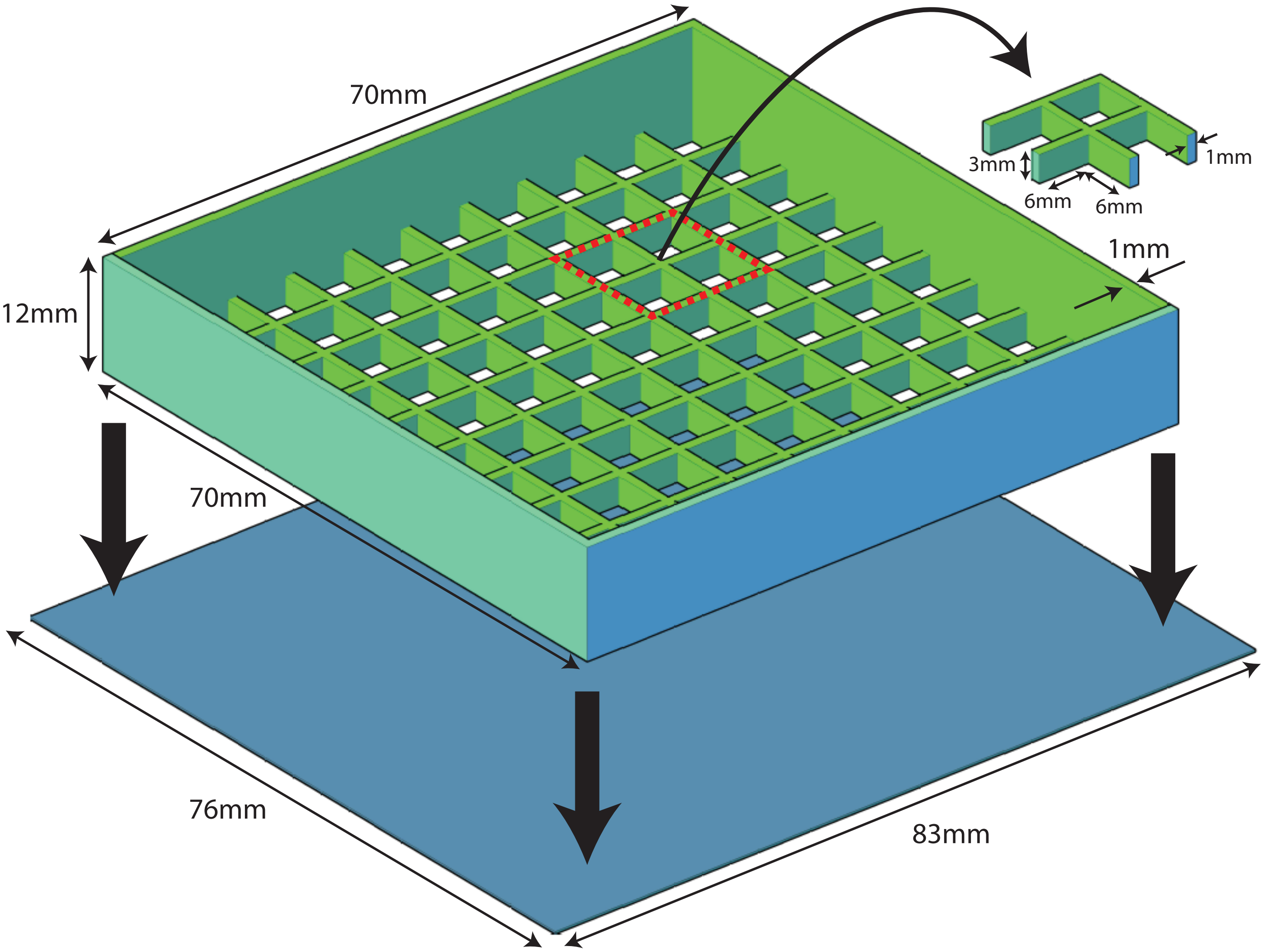

Supplementary Figure 1. Design of imaging chambers for 3D printing.

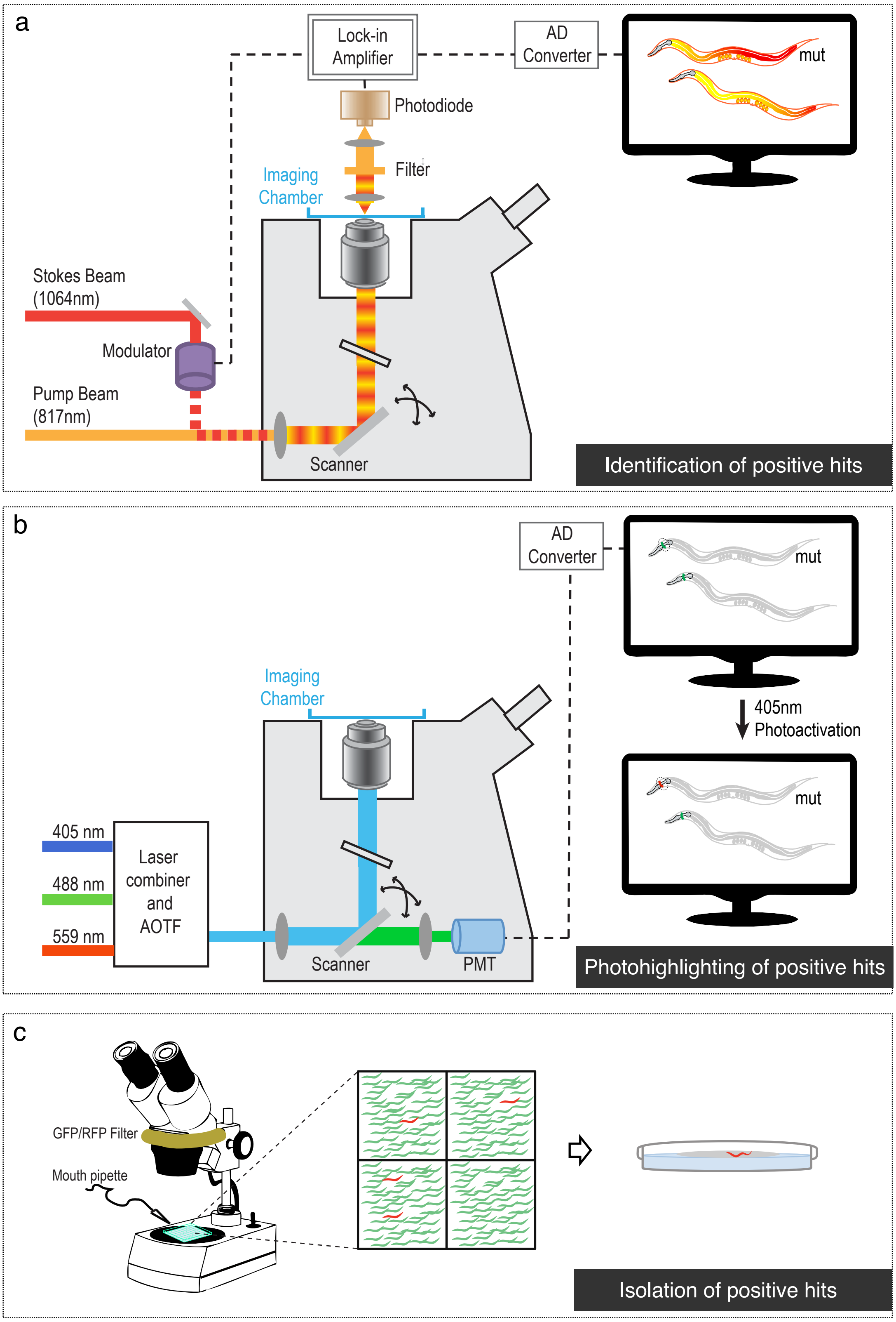

Supplementary Figure 2. Experimental scheme of high-throughput genetic screening.

(**a**) Diagram of the optical components for SRS microscopy built on a laser scanning confocal microscope. (**b**) Diagram of the conventional confocal imaging components on the same microscope used for photo-highlighting. PMT, photomultiplier tube. AOTF, acousto-optic tunable filters. (**c**) Recovery of photo-highlighted positive hits under a fluorescent dissection scope.

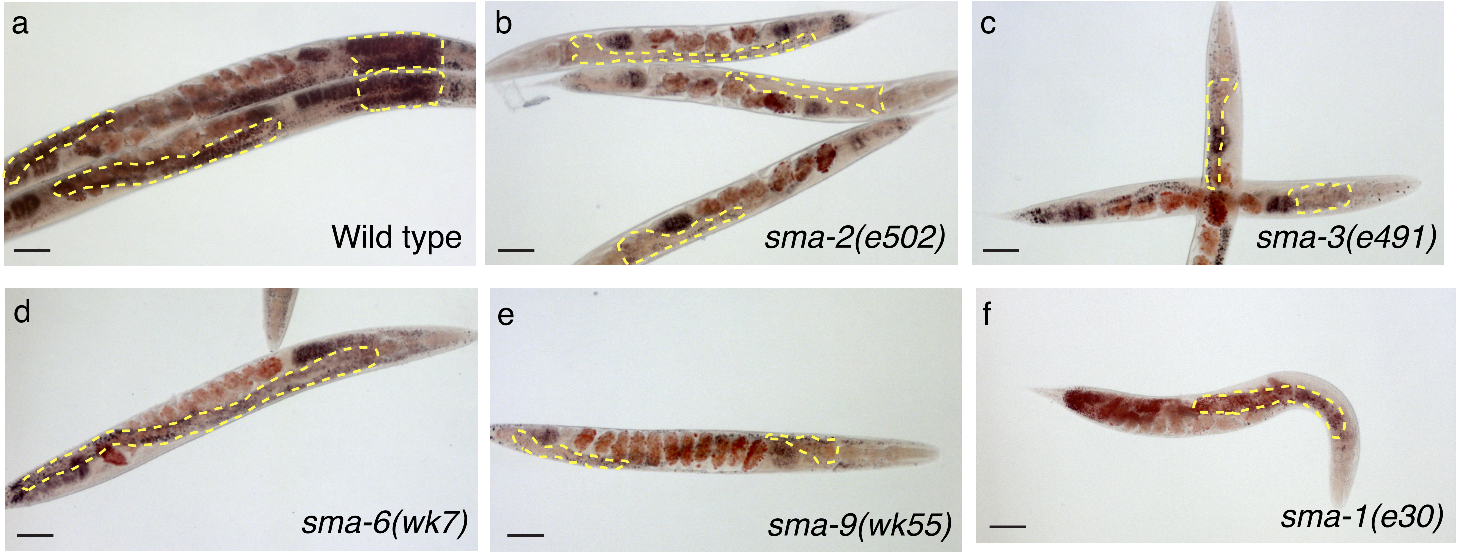

Supplementary Figure 3. Oil Red O staining images of *sma* mutants.

Representative images show lipid content levels in 1-day-old adult worms stained with Oil Red O. Yellow dashed lines indicate intestine areas. Scale bar=50 μm. Compared to wild type controls (**a**), lipid content levels are reduced in the *sma* mutants of the BMP signaling pathway (**b**-**e**), but not in the non-BMP *sma* mutant (**f**).

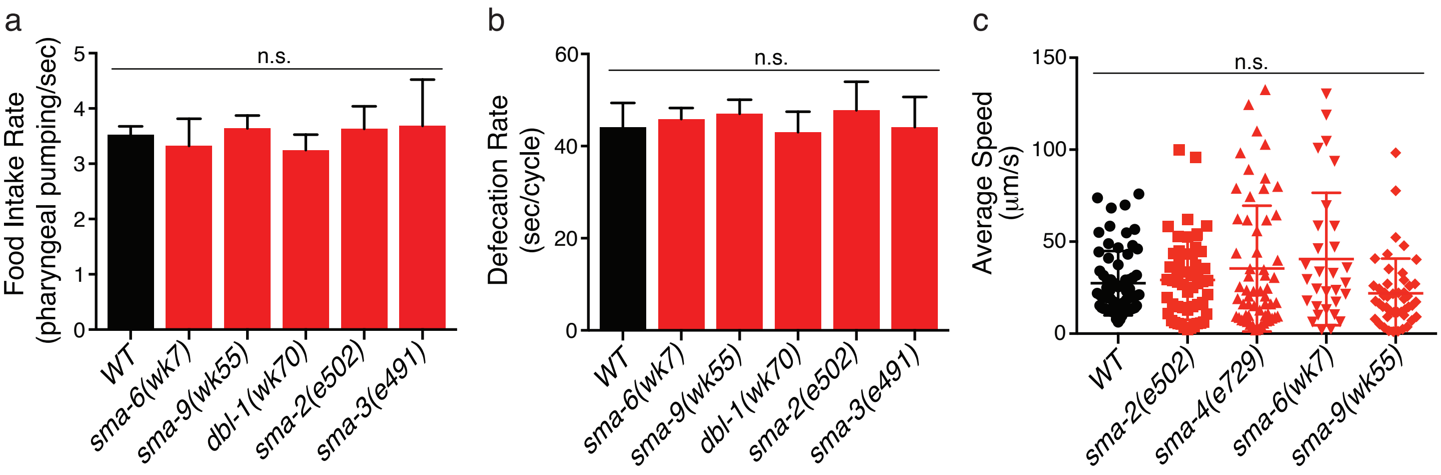

Supplementary Figure 4. Physiological measurement of sma/BMP pathway mutants.

Compared to wild type (WT) worms, the *sma* mutants show similar food intake rate (**a**, n=12), defecation interval (**b**, n=10), and locomotion activity (**c**, n=66 for WT, n=53 for *sma-2*, n=59 for *sma-4*, n=30 for *sma-6*, n=47 for *sma-9*). Error bars represent means±s.d., n.s. $p>0.05$ by one-way ANOVA.

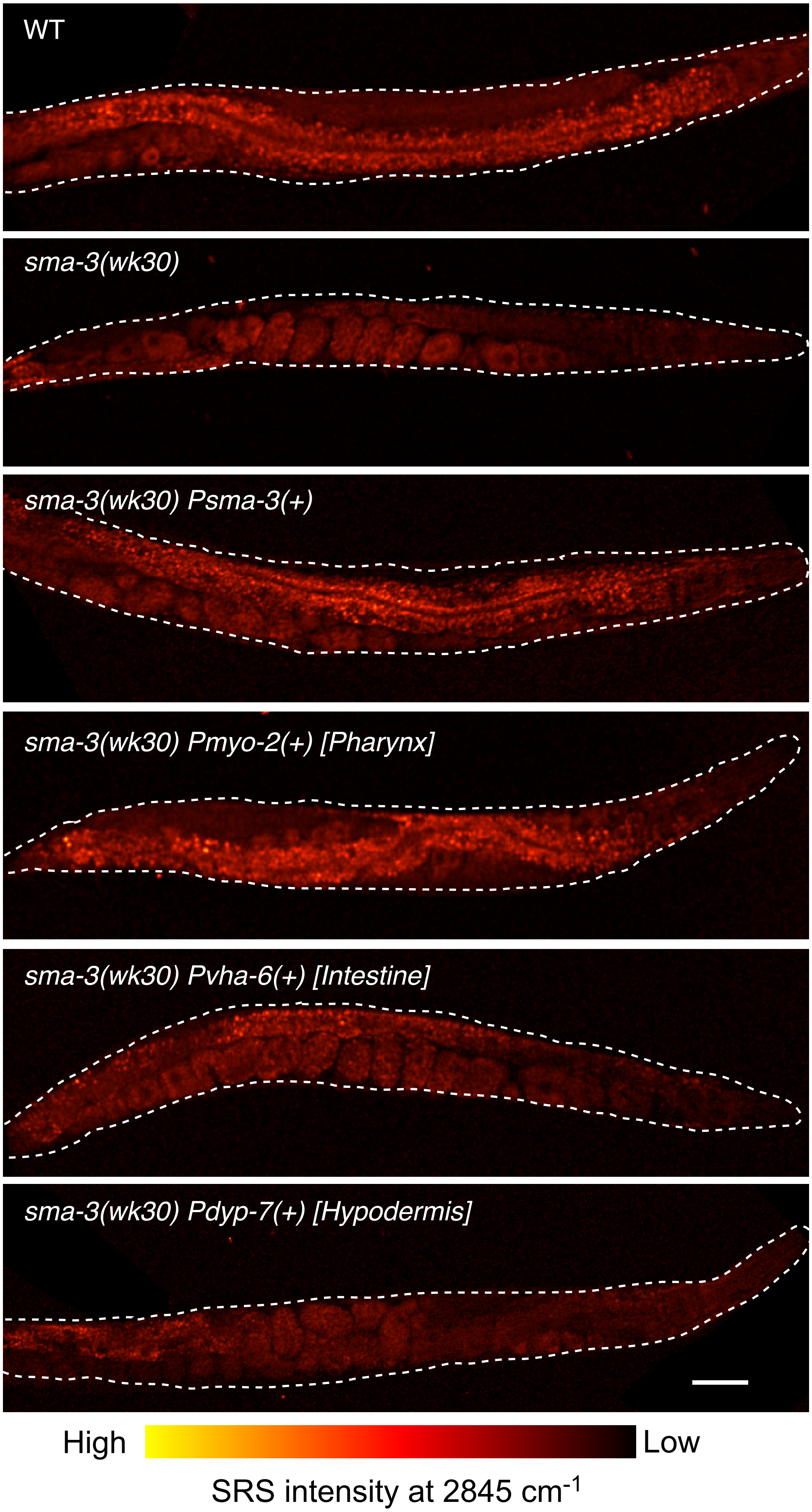

Supplementary Figure 5. Representatvie SRS images show lipid contents in wild-type, *sma-3* mutant, and *sma-3* tissue specific rescue strains.

Quantification results are shown in Fig. 3e. Dashed lines indicate the boundary of worms. Scale bar=50 μm.

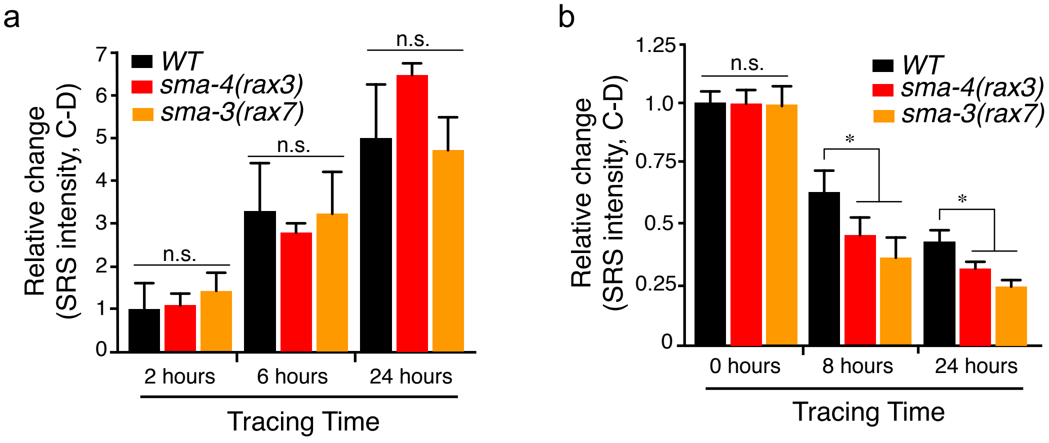

Supplementary Figure 6. Another iSRS replicate results reveal the regulation of lipid mobilization by BMP signaling.

(**a**) The BMP/sma mutants do not affect lipid synthesis, n.s. $p>0.05$, $n=5$.
(**b**) The BMP/sma mutants accelerate lipid mobilization, n.s. $p>0.05$, * $p<0.05$, $n=5$.
In all bar charts, data represent mean ± s.d., p value is determined by a Student's t-test (unpaired, two-tailed).

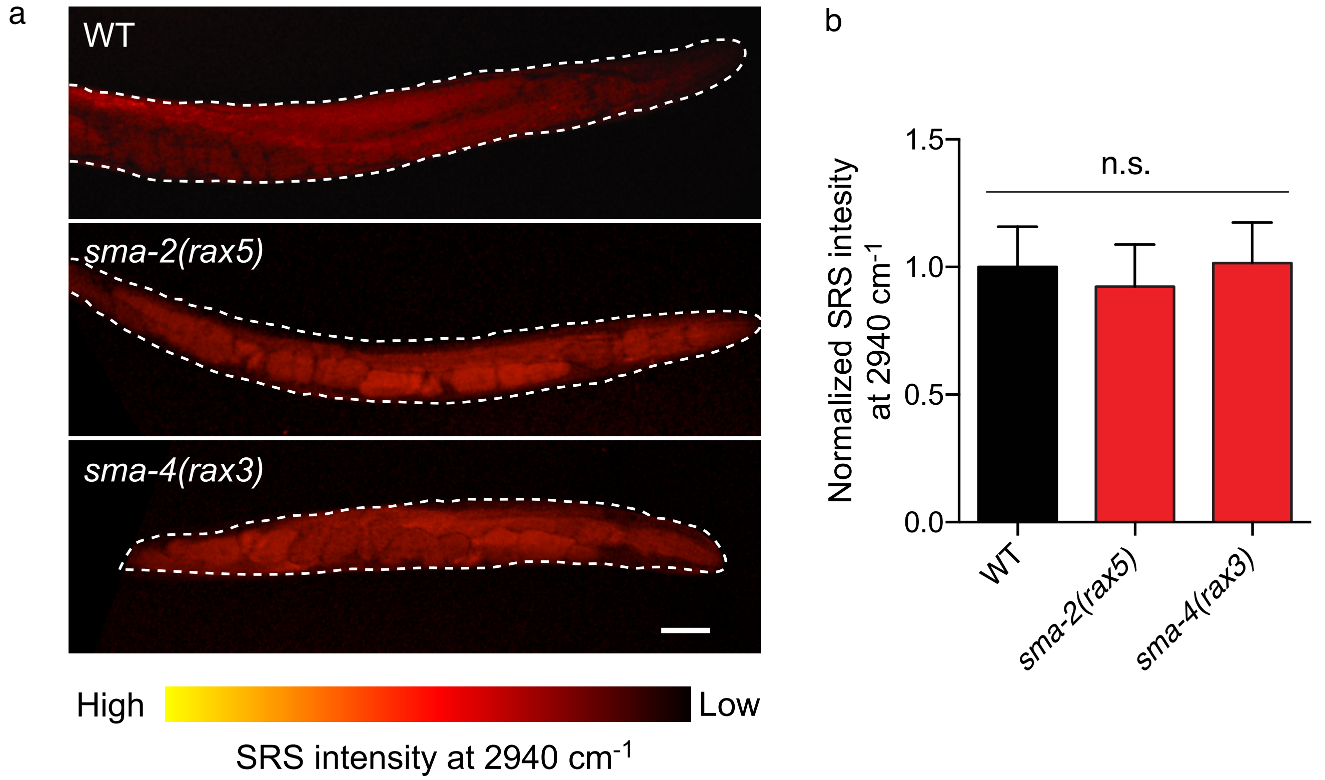

Supplementary Figure 7. SRS imaging at 2940 cm^{-1} reveals unchanged protein levels in *sma* mutants.

(**a**) Example SRS images at 2940 cm^{-1}. Dashed lines indicate the boundary of animals. Scale bar=50 μm.
(**b**) Quantification of SRS signal intensity at 2940 cm^{-1}. n.s. p>0.05 Student's *t*-test, n=43 for WT, n=25 for *sma-2*, n=44 for *sma-4*.

Supplementary Table 1. qPCR primers

acs-2 FWD	TTCGACCGGATGAGCCAGTAAACA
acs-2 REV	GTTGTTGTTCAGCCCGAAATGGGT
cpt-4 FWD	CAGAAACATCGCCAGTCTCA
cpt-4 REV	TCCATCCACTGACATCACAAA
acs-18 FWD	TGTGGCTCCAGATCTCACAG
acs-18 REV	GATCTGGGACAACGATTGCT
act-1 FWD	GCTGGACGTGATCTTACTGATTACC
act-1 REV	GTAGCAGAGCTTCTCCTTGATGTC
rpl-32 FWD	AGGGAATTGATAACCGTGTCCGCA
rpl-32 REV	TGTAGGACTGCATGAGGAGCATGT
